# The effects of ondansetron on diabetes and high-fat diet-induced liver disease: a critical role for protein tyrosine phosphatase 1B

**DOI:** 10.3389/fphar.2025.1565628

**Published:** 2025-04-28

**Authors:** Fawad Naeem, Maryam Aqeel, Muhammad Ammar Zahid, Mustafeez Mujtaba Babar, Fawad Ali Shah, Abdelali Agouni, Sohaib Zafar Malik

**Affiliations:** ^1^ Riphah Institute of Pharmaceutical Sciences, Riphah International University, Islamabad, Pakistan; ^2^ Shifa College of Pharmaceutical Sciences, Shifa Tameer-e-Millat University, Islamabad, Pakistan; ^3^ Department of Pharmaceutical Sciences, College of Pharmacy, QU Health, Qatar University, Doha, Qatar; ^4^ Department of Pharmacology and Toxicology, College of Pharmacy, Prince Sattam bin Abdulaziz University, Al-Kharj, Saudi Arabia

**Keywords:** ondansetron, PTP1B, diabetes, high-fat diet-induced obesity, non-alcoholic fatty liver disease

## Abstract

**Introduction:**

The escalating prevalence of diabetes and non-alcoholic fatty liver disease (NAFLD) has intensified the search for effective therapeutic interventions. The current study investigates the potential of ondansetron, a Food and Drug Administration (FDA)-approved drug for conditions like nausea and vomiting, as a novel treatment option for these metabolic disorders.

**Methods:**

A multifaceted approach, encompassing computational analyses, in vitro enzyme inhibition assays, and in vivo experiments in a high-fat diet (HFD)-induced disease model in rats were employed.

**Results:**

Computational studies, including pharmacophore modeling, molecular docking, and molecular dynamics (MD) simulations, revealed the strong binding affinity of ondansetron to the allosteric site of protein tyrosine phosphatase 1B (PTP1B), a key regulator of insulin and lipid homeostasis. The in vitro enzyme inhibition assay further confirmed ondansetron’s ability to directly inhibit PTP1B activity. Animal experiments demonstrated ondansetron’s antihyperglycemic effects, reducing blood glucose levels and improving insulin sensitivity in HFD-fed rats. The drug also exhibited hepatoprotective properties, mitigating liver damage and improving tissue architecture. Additionally, ondansetron’s anti-inflammatory and antioxidant activities were evident in its ability to reduce pro-inflammatory markers and oxidative stress in the liver.

**Discussion:**

These therapeutic effects position ondansetron as a promising candidate for further investigation in clinical settings for the treatment of diabetes and NAFLD and, hence, support the use of the drug repurposing approach for addressing the growing burden of metabolic diseases.

## Introduction

Diabetes mellitus (DM), a metabolic syndrome, characterized by elevated blood glucose levels and defective metabolism of carbohydrates, proteins, and lipids, is becoming one of the most prevalent clinical conditions. Although type 1 DM tends to be genetically induced, more than 80% of all diabetes cases fall are of type 2 DM (T2DM). The dysfunction of beta cells in the pancreas leads to irregularities in insulin levels, abnormal blood glucose concentration, insulin insufficiency, and insulin resistance (American Diabetes Association, 2010). Of the various molecular mechanisms regulating blood glucose levels, leptin and insulin pathways emerge as the most important ones. Protein tyrosine phosphatase 1B (PTP1B) forms an essential component of these pathways in which it acts as a negative regulator of both leptin and insulin signaling. Dephosphorylation of key metabolic constituents like insulin receptor (IR) and JAK2 results in the inhibition of the signaling mechanism that is essential for the normal functioning of glucose metabolism (Tonks, 2013). Mice fed a high-fat diet (HFD), which had limited expression of PTP1B in the hypothalamic region, showed resistance to obesity and enhanced sensitivity to insulin/leptin (Picardi et al., 2008). Increased insulin sensitivity was also reported in PTP1B-knockout mice (Elchebly et al., 1999; Klaman et al., 2000). It can be inferred that increased insulin sensitivity is observed upon PTP1B activity inhibition, highlighting it as a potential target for T2DM and obesity (Villamar-Cruz et al., 2021).

The role of PTP1B has been established in a similar metabolic condition, non-alcoholic fatty liver disease (NAFLD) as it is involved in the deposition of fat in the liver. Upon its inhibition, insulin sensitivity improves, allowing for more effective fat storage. Moreover, apoptosis of hepatocytes is also avoided by limiting PTP1B enzymatic activity, which proficiently accounts for the development of NAFLD. Due to the inhibition of PTP1B activity, the preventive action of signaling pathways that support cell survival is improved, resulting in compensatory mechanisms like inflammation and liver damage associated with NAFLD. A decrease in the production of PTP1B is also associated with a better mitochondrial function as improved oxidation of fat and reduced deposition in the liver are observed under normal conditions (Chen et al., 2015).

Regulation of PTP1B leads to effective inhibition of various inflammatory processes. The primary feature of NAFLD is persistent inflammation, which, along with increased fat deposition, further results in a condition called non-alcoholic steatohepatitis (NASH) (Pan et al., 2022). The role of PTP1B activity inhibition has been identified in hepatocyte proliferation and liver regeneration, which could, hence, be instrumental in treating NAFLD patients (Chen et al., 2015). Moreover, improvement in abnormal conditions like inflammation, cellular apoptosis, and insulin resistance advocates for the diverse pharmacological activities of PTP1B, thus presenting it as an attractive therapeutic target for conditions like NAFLD (Pan et al., 2022).

Development of drugs against this identified target can yield effective pharmacological and therapeutic outcomes for the treatment of NAFLD and other related conditions. However, the conventional drug discovery process is laborious and expensive. Time and cost related to the drug discovery can be significantly reduced by employing virtual high-throughput screening techniques using FDA-approved molecules. As the effectiveness and safety profiles of these molecules have already been established, such pharmacoinformatics approaches including molecular docking, pharmacophore screening, and molecular dynamics (MD) simulations can be used for repurposing these molecules for decreasing the time from the bench to bedside (Ou-Yang et al., 2012). After establishing their effectiveness *in silico*, these molecules can be tested through *in vitro* and *in vivo* approaches to establish their candidature for drug development.

A variety of compounds have been identified, developed, and categorized as PTP1B inhibitors. However, there has been limited success in clinical trials due to their limited specificity, effectiveness, and low safety profile. The majority of these inhibitors bind to the conserved active site of PTP1B that has constraints like similarity in structure and strong charge, posing issues in selectivity and penetrating ability (Yang et al., 2021). In order to address this, developing allosteric inhibitors with broader pharmacological action and improving the flexibility with respect to the secondary binding site can be helpful.

This study aimed to perform virtual screening of the FDA-approved drug library against the PTP1B protein, targeting the allosteric binding site by employing a series of computational techniques, including pharmacophore modeling, molecular docking studies, and MD simulations. The top candidate identified in the study, ondansetron, was subjected to further experimental procedures to establish its candidature. Ondansetron, a well-established and clinically employed antagonist of serotonin (5-HT_3_) receptors, is used as an anti-emetic agent mainly in cancer chemotherapy and surgery (Wilde and Markham, 1996). In order to establish the selectivity of the molecule, an *in vitro* enzyme inhibition assay specific for PTP1B was employed. Thereafter, a rat model of NAFLD induced by a HFD was established and treated with the drug molecule. Hematological and metabolic profiles of the animals were assessed to establish the effect of test molecules on lipid metabolism, oxidative stress, inflammation, and insulin sensitivity.

## Materials and methods

### Chemicals

All the chemicals used in this study were purchased from authentic and verified sources. Ondansetron was purchased from Sigma CO, MERK. Streptozotocin was purchased from Sigma-Aldrich Co., LLC, United States of America. Antibodies anti-phosphorylated nuclear factor kappa B (NF)-κB, anti-tumor necrosis factor (TNF)-α, and anti-cyclooxygenase (COX-2) were purchased from Abcam UK.

### Pharmacophore modeling and screening

The pharmacophore model was developed using the ligand–protein complex from PDB:1T48. Energy minimization of compounds was performed with LigPrep2 (Schrödinger, LLC, 2021) using the OPLS_2005 force field, adding hydrogens and generating low-energy conformations and tautomers, with the ionization state set to neutral. The PHASE module in Maestro software created the pharmacophore model, identifying six features: hydrophobic group (H), hydrogen bond acceptor (A), negatively charged group (N), hydrogen bond donor (D), aromatic ring (R), and positively charged group (P). The FDA-approved drug database was screened against this model, with four pharmacophore attributes set as “must match.” The PHASE module of Schrödinger software was used, which included different steps in which the protein having PDB ID: 1T48 was downloaded from the Protein Data Bank. A local database was constructed from http://www.drugbank.ca, which carried all the necessary information regarding various aspects like experimental, investigational, and approved drug profiles. This study primarily focuses on the approved drugs with known structures and pharmacological activities. Out of 2,470 approved drugs present in the databank, our local database was created using the Schrödinger database path. A total of 50 ligand conformers were generated and minimized according to their database IDs. Screening was carried out against all these ligands, and pharmacophore hypothesis was selected against four to seven matches, and finally, the job was run.

### Molecular docking studies

Molecular docking studies were conducted using the Glide module within the Maestro software suite (Schrödinger Release 2024-3: Maestro, Schrödinger, LLC, New York, NY, 2024) to identify optimal ligand orientations within the binding site of the target protein, PTP1B (PDB ID: 1T48), and to calculate binding affinities through docking scores (Friesner et al., 2004). Protein preparation wizard in Maestro was used to optimize the protein, including homology modeling of a missing loop (residues 283–290), hence ensuring that the structure was ready for docking simulations. Ligands were sourced from DrugBank and prepared using LigPrep (Schrödinger Release 2024-3: LigPrep, Schrödinger, LLC, New York, NY, 2024), which generated appropriate ionization states, tautomers, stereoisomers, and 3D conformations. A receptor grid was created to define the active site for ligand docking, taking into account the surrounding residues of the binding pocket. The prepared protein and ligands were docked using the Standard Precision (SP) docking algorithm in Glide, which identified favorable ligand poses and calculated corresponding docking scores for ranking and selection based on predicted binding affinities in kcal/mol.

### Molecular dynamics simulation and binding free energy estimation

To investigate the dynamic behavior of complexes obtained from molecular docking, MD simulations were conducted using the AMBER23 simulation package and AMBERtools23 with the FF19SB force field (Tian et al., 2020; Case et al., 2023). Ligand topology was generated via the antechamber tool using the general AMBER force field 2 (GAFF2). Each complex was solvated in a rectangular box with an “optimal point charge” (OPC) of 15.0 Å on each side, and sodium ions were added for system neutrality. The systems were relaxed using steepest descent and conjugate gradient techniques for energy minimization, followed by heating to 300 K and equilibrating for 2 ns under mild restraints. A production run of 500 ns was then performed for each complex. Temperature regulation was achieved with Langevin thermostats, while long-range electrostatic interactions were managed using the particle-mesh Ewald (PME) method. The SHAKE algorithm was used to maintain covalent bonds. To enhance simulation speed, we used the GPU-accelerated pmemd.cuda approach. Trajectory processing was facilitated using the CPPTRAJ and PTRAJ modules. The Prime MM-GBSA module in Schrödinger was used to calculate the binding free energies (BFEs).

## *In vitro* studies

### PTP1B enzyme inhibition assay

The PTP1B inhibition assay was performed using the PTP1B Inhibitor Screening Assay Kit (#ab139465, Abcam, Cambridge, UK) adopting an established method (Xie et al., 2022). A phosphate standard curve was created using the PTP1B assay buffer and 100 µM of phosphate standard solutions. For the assay, 35 µL of PTP1B assay buffer, 5 µL of PTP1B diluent, and different concentrations of suramin (0–500 µM) were mixed, totaling 50 µL. Control wells contained 40 µL of buffer and 10 µL of DMSO, while time zero wells had 35 µL of buffer, 10 µL of DMSO, and 5 µL of the diluent. After adding 50 µL of the substrate, plates were incubated at 37°C for 30 min. The reaction was terminated with 25 µL of the red assay agent, and absorbance was measured at 620 nm. Optical density values were converted to nmol of phosphate using the standard curve, and the percent activity of the enzyme was calculated.

### *In vivo* studies

The animal studies adhered strictly to protocols approved by the Research and Ethics Committee of the Riphah Institute of Pharmaceutical Sciences (REC-RIPS), under approval reference REC/RIPS/2021/021. All procedures conformed to the utmost ethical standards of animal welfare and research integrity. The experiments were conducted in accordance with institutional protocols and adhered to the *Guide for the Care and Use of Laboratory Animals* (NIH Publication No. 85-23, revised 2011), issued by the US National Institutes of Health.

The rat model of NAFLD caused by an HFD was established according to previously documented methodologies (Carreres et al., 2021). Male and female Wistar rats (300–350 g), aged between 12 and 16 weeks, were housed in groups of five, kept under a 12-h light/dark cycle (lights on from 8:00 to 20:00) at a controlled temperature of 23°C–25°C, with unrestricted access to water and food (dietary specifics are detailed in Supplementary Table S1). After an acclimatization phase, the rats were allocated into five groups (n = 5 per group): non-disease control (normal chow diet, 4 months), diseased control (HFD, 4 months), diseased + 10 mg/kg ondansetron (28 days), diseased + 20 mg/kg ondansetron (28 days), and diseased + 100 mg/kg metformin (28 days). These experimental groups were used to assess the impact of the treatments on HFD-induced NAFLD and NASH.

### Determination of glucose, insulin, and lipid levels

After a 4-week treatment, an oral glucose tolerance test (OGTT) was conducted on overnight-fasted rats (Tang et al., 2018). They received 25% dextrose (2 g/kg), and blood glucose levels were measured at 0-, 30-, 60-, 90-, and 120-min post-dose using an Accu-Chek glucometer (Accu-Chek, UK). Fasting serum insulin and glucose levels were determined via cardiac puncture under terminal diethyl ether anesthesia, with blood collected in EDTA tubes and centrifuged at 35,400 rpm for 10–20 min at room temperature. Serum lipid markers (cholesterol, triglycerides, LDL, and HDL) were analyzed using an Olympus AU-2700 auto-analyzer (Olympus, Japan).

### Liver enzymes

Liver function tests were performed using an Olympus AU-2700 auto-analyzer (Olympus, Japan) with commercially available kits to measure alanine aminotransferase (ALT), aspartate aminotransferase (AST), and total bilirubin (TB) in separated serum.

### Gross and histopathological examination of the liver

After 28 days of treatment, rats were euthanized, and their livers were extracted, rinsed with normal saline, and placed in Petri dishes for physical examination. The livers’ color, size, and evidence of inflammation or damage were compared among normal, disease, and treated groups. Liver tissues were then used to prepare histological slides and for homogenization.

Hematoxylin and eosin (H&E) staining was performed using a previously reported method (Shah et al., 2018). Liver tissue sections of 3–5 µm were deparaffinized with xylene (100%), rehydrated with graded ethanol (100%, 95%, and 70%), rinsed in distilled water, and stained with hematoxylin for 10 min. After rinsing under tap water, slides were treated with 1% HCl and 1% ammonia water, followed by eosin staining for 10 min. Thereafter, slides were rinsed, air-dried, dehydrated in ethanol (70%, 95%, and 100%), and cleared in xylene. Images were captured using an Olympus light microscope, focusing on hepatocyte characteristics. TIF images were standardized for intensity and analyzed using ImageJ software [National Institutes of Health (NIH), USA].

### Immunohistochemical analysis

Immunohistochemical analysis was performed on paraffin-embedded, formalin-fixed tissue sections (Shah et al., 2019). Sections were deparaffinized and rehydrated, and antigens were retrieved enzymatically. Endogenous peroxidase activity was blocked with 3% hydrogen peroxide in methanol, and non-specific binding was inhibited using 5% normal goat serum with 0.1% Triton X-100. Primary antibodies against TNF-α, COX-2, and NF-κB (1:100) were applied overnight at 4°C. After rinsing with PBS, biotinylated secondary antibodies (1:50) were applied for 1 h, followed by the ABC Elite kit. Sections were stained with DAB, counterstained with hematoxylin, and analyzed using ImageJ software. Data were expressed as relative integrated density compared to saline control.

### Inflammatory mediators in liver tissue

Liver tissues (50–70 mg) were homogenized with PBS buffer, and the supernatant was collected after centrifugation at 4,000 rpm for 20 min. ELISA kits provided by Elabscience China, the NF-κB ELISA kit (Cat #: E-EL-RO674), and the TNF-α ELISA kit (Cat #: E-EL-R0019) with specific antibodies for NF-κB, TNF-α, and COX-2 were used according to the manufacturer’s instructions. Protein samples were tested in 96-well plates, and absorbance values were measured using a microplate reader. Concentrations were calculated in picograms per milliliter (pg/mL) and normalized to total protein content in picograms per milligram (pg/mg of total protein). All samples were analyzed in triplicates.

### Antioxidant enzymes and peroxidation in liver tissue

To assess oxidative damage and the effects of the test drug, liver tissue samples were homogenized in 0.1 M sodium phosphate buffer (pH 7.4) containing phenylmethylsulfonyl fluoride (PMSF) and then centrifuged at 4,000 g for 10 min at 5°C. Glutathione (GSH) levels were measured by adding 40 μL DTNB in 100 μL of sodium phosphate buffer to 6.6 μL of the supernatant in a 96-well plate, with the absorbance read at 412 nm after 10 min, and GSH values were expressed in μmol GSH/mg of the sample (Imran et al., 2020). Glutathione S-transferase (GST) activity was assessed by adding 10 μL of the tissue supernatant to a mixture of 10 μL of GSH (5 mM) and 10 μL of 2,4-dinitrobenzene (CDNB) (1 mM) in 100 μL of phosphate buffer, recording at 340 nm for 5 min, and the activity was expressed as μmoles of CDNB conjugated/min/mg of protein (Al Kury et al., 2019). Catalase (CAT) activity was estimated by adding 20 μL H_2_O_2_ to 10 μL of the supernatant and measuring the absorbance every 30 s for 2 min to assess the decomposition of H_2_O_2_ per mg of the homogenate (Weydert and Cullen, 2010).

Lipid peroxidation was assessed by measuring thiobarbituric acid reactive substances (TBARSs) using a colorimetric technique (Iqbal et al., 2020). The assay mixture included 36 μL of buffer (pH 7.4), 25 µL of the supernatant, 1.25 µL FeCl_2_, and 12.5 µL of 100 mM ascorbic acid. This mixture was incubated at 37°C for 60 min. The reaction was terminated by adding 62.5 µL of 10% trichloroacetic acid (TCA) and 62.5 µL of 0.66% thiobarbituric acid (TBA), followed by a 20-min water bath incubation, cooling, and centrifugation at 3,000 g for 10 min. The absorbance was measured at 535 nm.

### Statistical analyses

Both descriptive and inferential statistics were used. GraphPad Prism 8 was used as a statistical tool. For determining the central tendency and deviation, mean ± SEM was used. One-way ANOVA was used for hypothesis testing while considering *p*-value <0.05 as significant.

## Results

### Pharmacophore modeling and screening

**FIGURE 1:**
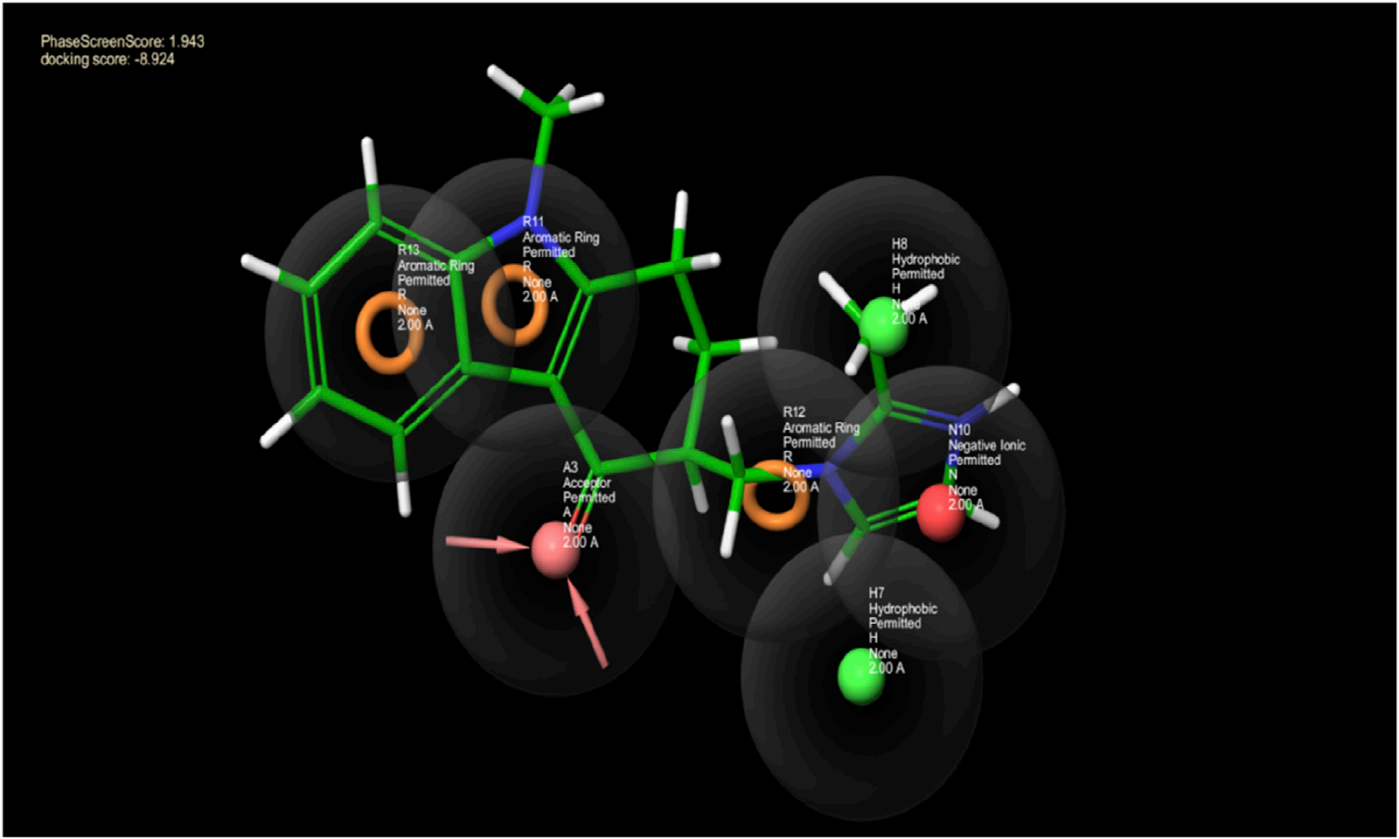
This picture depicts the spaces between pharmacophore sites pertaining to the pharmacophore hypothesis of the protein and ligand complex. The hydrogen bond acceptor having a lone pair of electrons is indicated by the arrows in the pink sphere, whereas the aromatic ring is highlighted by the orange rings, and the green sphere represents the hydrophobic species. The negative ion is indicated by the red sphere. The green ball and stick model reflect the alignment of the pharmacophore model with ondansetron.

The model was constructed based on a 7-point common pharmacophore hypothesis, denoted as ANHHRRR (Figure 1). This hypothesis encompasses various chemical features crucial for ligand–receptor interactions. It comprises one hydrogen bond acceptor (A3), located at a specific position within the pharmacophore, enabling the formation of hydrogen bonds with complementary donor groups on the target receptor. Additionally, there are two hydrophobic regions (H7 and H8), which contribute to van der Waals interactions and play a role in the binding affinity. The negative ion (N10) represents an electronegative site that can interact with positively charged regions on the receptor. Moreover, three aromatic rings or hydrocarbon chains (R11, R12, and R13) are incorporated into the hypothesis, providing additional hydrophobic interactions and potential for pi-stacking. The FDA-approved drug database was screened against this hypothesis, and compounds were ranked according to the phase screen score. Ondansetron has a phase screen score of 1.943, making it a top hit in the screening process. The phase screen scores of the top 20 compounds are presented in Supplementary Table S1.

### Molecular docking studies

**FIGURE 2:**
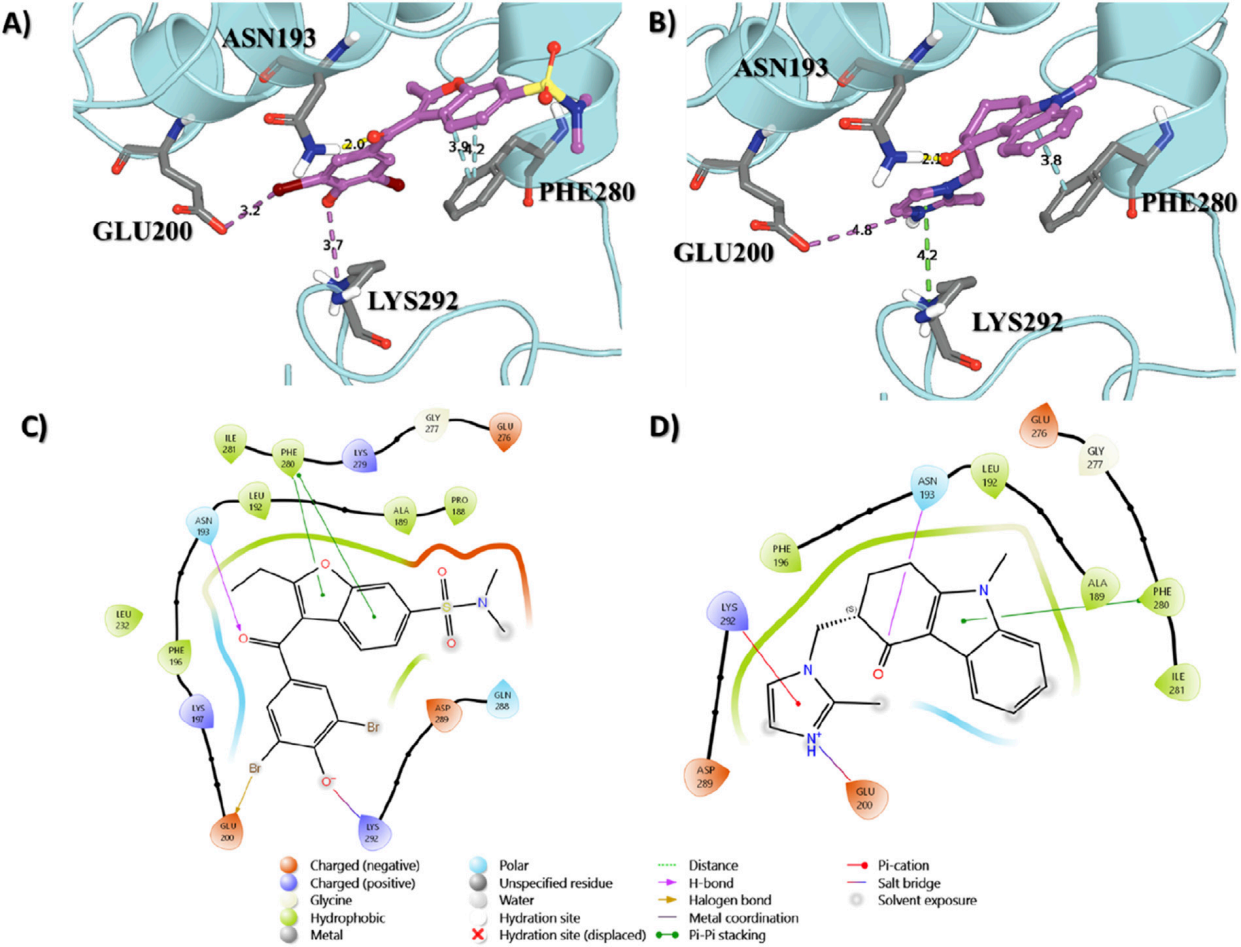
Binding interactions of the native ligand **(A,C)** and ondansetron **(B,D)** with the protein structure. Key residues involved in the binding process are labeled, showing their proximity to the ligand molecules.

The interaction between the ligand proteins and two distinct molecules, the native ligand in 1T48 and ondansetron, reveals critical binding interactions through hydrogen bonds, van der Waals forces, and pi–pi interactions. As represented in Figure 2, significant bonds were observed between the 1T48-reported ligand and the proteins at ASN193 (2.0 Å), LYS292 (3.7 Å), GLU200 (3.2 Å), and PHE280 (3.9 Å). Similar binding patterns were observed for ondansetron, with a hydrogen bond with ASN193 (2.2 Å), a salt bridge with GLU200 (4.8 Å), pi -cation interaction with LYS292 (4.2 Å), and a pi -pi interaction with PHE280 (3.8 Å) as indicated in Figure 2. The interaction lengths suggest a stable binding confirmation with key residues positioned effectively for ligand interaction. These interactions and the shape complementarity of ondansetron contribute to the docking score of −8.92, making it among the top-ranking compounds. The docking scores of the top 20 screened compounds are present in Supplementary Table S2.

### MD simulation and total binding free energy estimation

**FIGURE 3:**
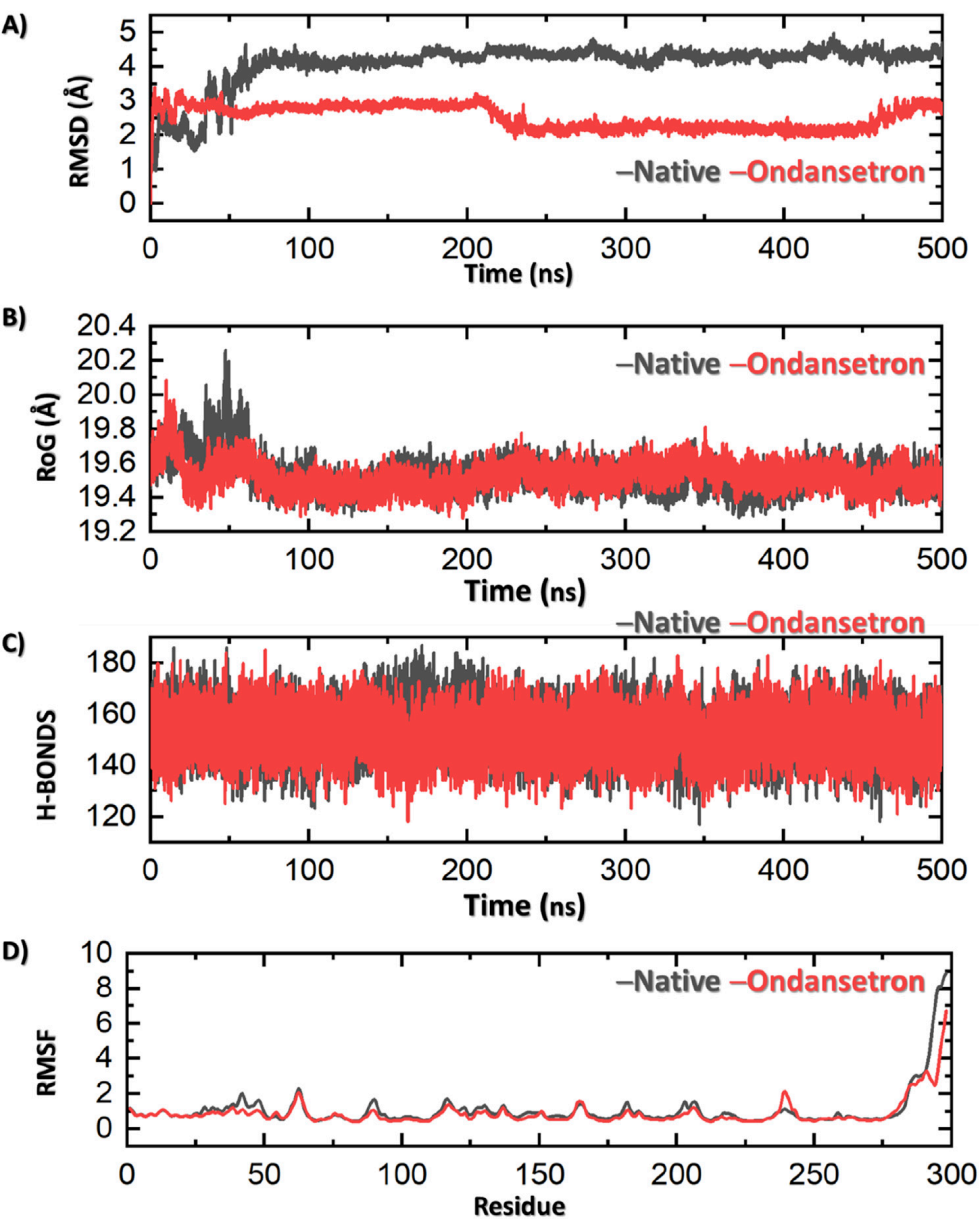
Results of MD analysis shows ondansetron + bound protein (represented in red) and native ligand + protein (represented in black). **(A)** Stability of the structure by RMSD. **(B)** Compactness via Rg. **(C)** Number of hydrogen bonds indicating molecular interactions. The fluctuations in various residues of the protein along the simulation are represented in **(D)** by RMSF. Each figure showcases the variations in dynamic behavior between the ondansetron–protein complex and the native protein in a simulation of 500 nanoseconds.

The MD simulation was conducted to evaluate the relative binding stability and structural characteristics of the 1T48 protein system complexed with either the native ligand or ondansetron. Several key parameters were analyzed, including root mean square deviation (RMSD), radius of gyration (Rg), hydrogen bond formation, and root mean square fluctuation (RMSF). These parameters provide insights into the conformational stability and interactions of each ligand within the protein–ligand complex. RMSD is a key indicator of the overall stability and convergence of the protein–ligand complex during the simulation. The first panel in Figure 3 illustrates that the RMSD values for the protein when complexed with ondansetron (red) exhibit significantly lesser fluctuations when compared to the native ligand (black). Although the native ligand complex stabilized around an RMSD value of approximately 4.0 Å, indicating more significant deviations from the initial structure, the ondansetron complex achieved lower RMSD values, stabilizing at approximately 2.5 Å.

A much stable and consistent interaction is observed between the protein and ondansetron, which indicates that the structural integrity of this complex is far better than that of the native ligand. The overall shape of the protein structure during MD simulation and the compactness of the structure of protein can be measured using the Rg values. Figure 3 (second panel) indicates that the drug–protein complex of ondansetron produces a lower and stronger Rg value of 19.4 Å in comparison with that of the native ligand, that is, 19.7 Å. This slight compactness of the ondansetron–protein complex advocates for better stability and lesser ability of the protein to be altered. Hence, the overall stability of this complex is observed.

Hydrogen bonds play a crucial role in the maintenance of the protein–ligand interaction and overall complex stability. In the third panel of Figure 3, the hydrogen bond analyses indicate that the ondansetron-bound complex has a consistently comparable average number of hydrogen bonds as compared to the native ligand-bound complex (158 vs. 156), further suggesting its stability within the protein-binding site. RMSF measures the flexibility of individual amino acid residues during the simulation, providing insights into the dynamic behavior of the protein. The fourth panel of Figure 3 reveals that the RMSF values of the residues in the presence of ondansetron (red) are generally lower or comparable to those in the presence of the native ligand (black). Slight deviations in RMSF values are observed in particular regions of the protein, where ondansetron complexation resulted in reduced fluctuations. This indicates that binding to ondansetron may induce rigidity in these protein regions, which contributes to the stabilizing effect observed in other stability measures, such as RMSD and Rg. The total binding free energy (BFE) of the 1T48 native ligand was −59.73 ± 3.14 kcal/mol, while for ondansetron, it was estimated to be −51.40 ± 2.94 kcal/mol.

### *In vitro* PTP1B enzyme inhibition

**FIGURE 4:**
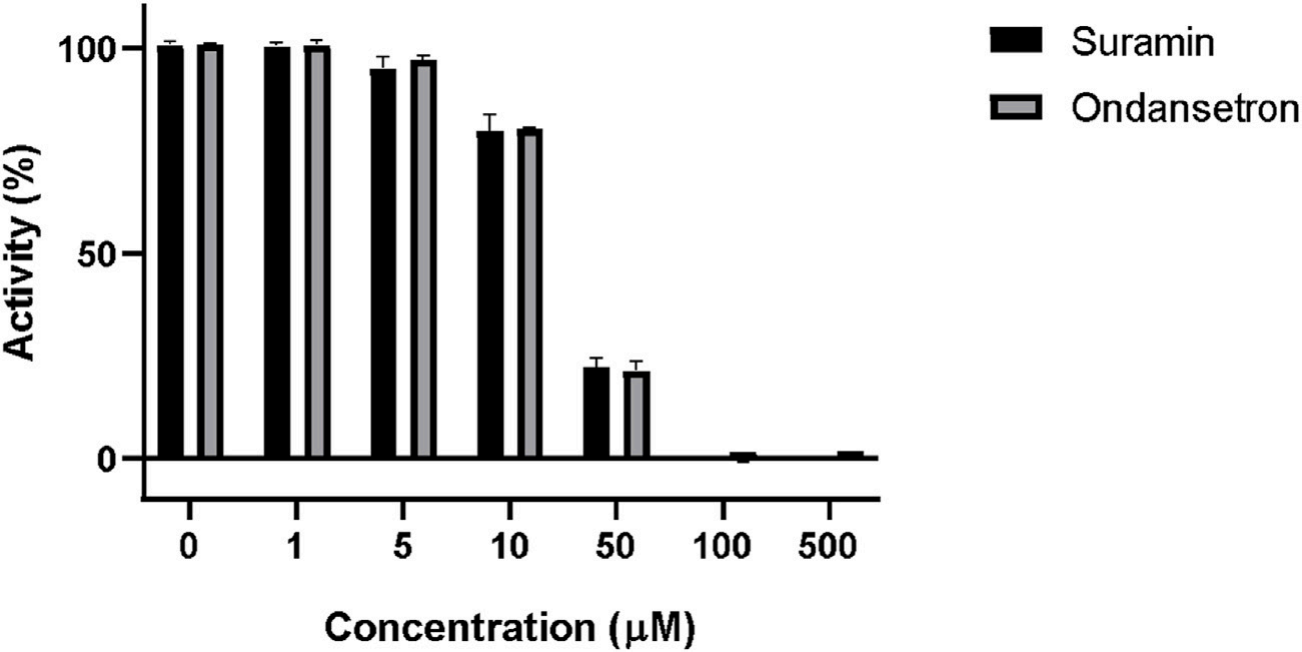
Inhibition of PTP1B enzymatic activity by suramin and ondansetron (gray bars) at different concentrations. The X-axis represents the increasing concentrations of each compound, while the y-axis shows the % activity of the PTP1B enzyme.

The percent activity of suramin and ondansetron against PTP1B in the enzyme inhibition assay is shown in Figure 4. The percent activities of the enzyme at 10 uM of suramin and ondansetron were 79.7% ± 4.0% and 80.3% ± 0.2%, respectively, while activities were reduced to 22.1% ± 1.3% and 21.2% ± 3.7% at 50 uM. A concentration of 100 uM of the compound completely blocked the enzymatic activity. Statistical analysis revealed no significant difference in the ability of ondansetron and suramin to inhibit PTP1B activity across various concentrations, indicating similar inhibitory capabilities. The variability in inhibition between the two groups was only 11%, highlighting minimal differences. A strong correlation between paired observations was evidenced by a correlation coefficient of 0.98 and a p-value of <0.01. In summary, ondansetron exhibits comparable potency to the positive control, suramin, in inhibiting the activity of the target protein.

### *In vivo* animal studies

**FIGURE 5:**
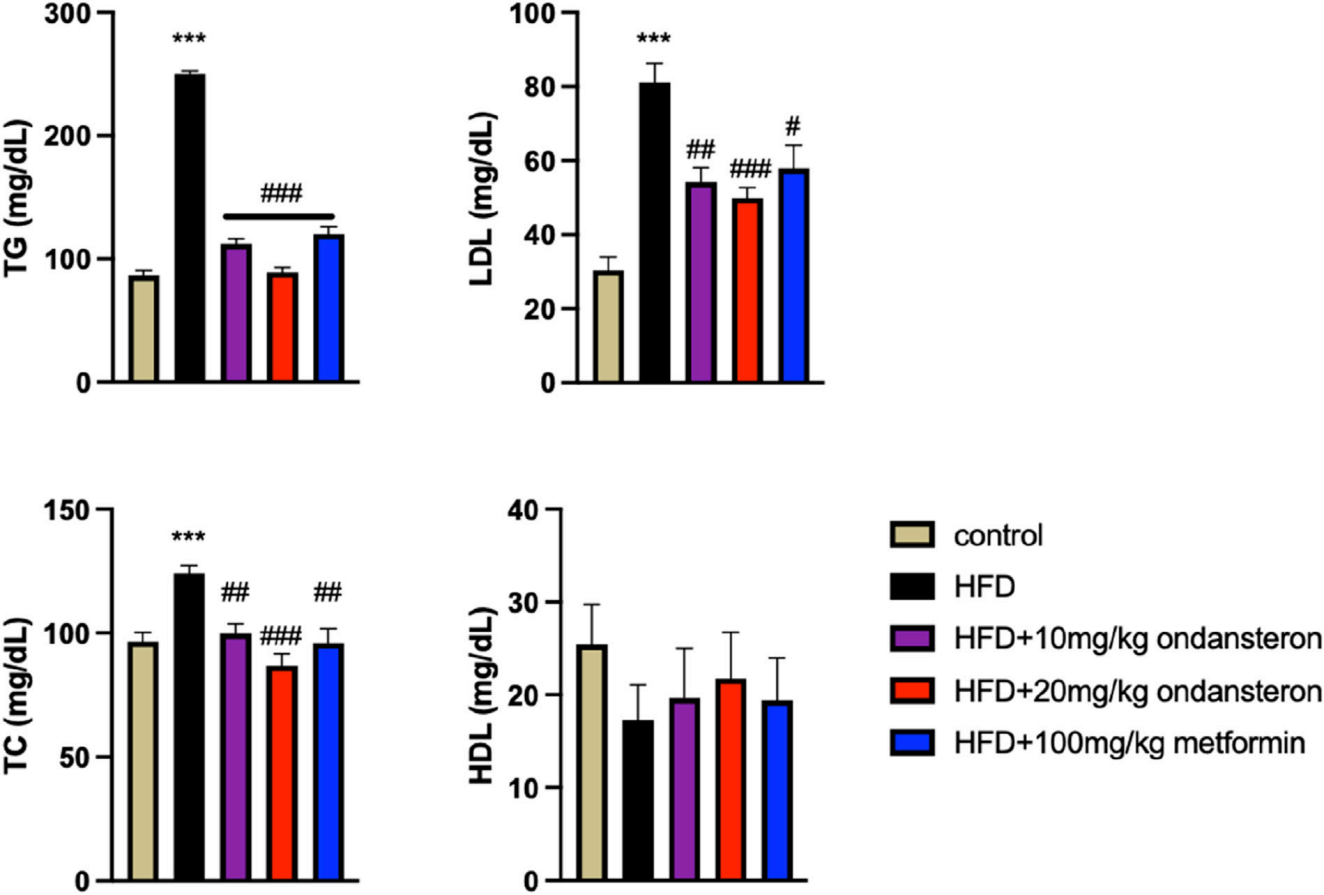
Impact of ondansetron and metformin on enhanced serum lipid contents (in serum TG, TC, LDL, and HDL cholesterol) caused by HFD. Both low and high doses of ondansetron and metformin reduced the circulating levels of TG, TC, and LDL in rats fed an HFD. No significant effect of the drugs was observed for HDL cholesterol. Data are presented as means *±* SEM. One-way ANOVA is used for the analysis of data, followed by a post hoc Bonferroni multiple comparison test using GraphPad Prism 5 software. The symbols * or # represent significant difference values p < 0.05, ** or ## represents p < 0.01, and *** or ### represents p < 0.001 values for significant differences. The symbol * shows a significant difference relative to control, and # shows a significant difference relative to the HFD group.

The administration of ondansetron (10 mg/kg and 20 mg/kg) and metformin (100 mg/kg) resulted in significant reductions in serum TG, TC, and LDL cholesterol levels in HFD-fed rats as represented in Figure 5.

### Determination of glucose, insulin, and lipid levels

**FIGURE 6:**
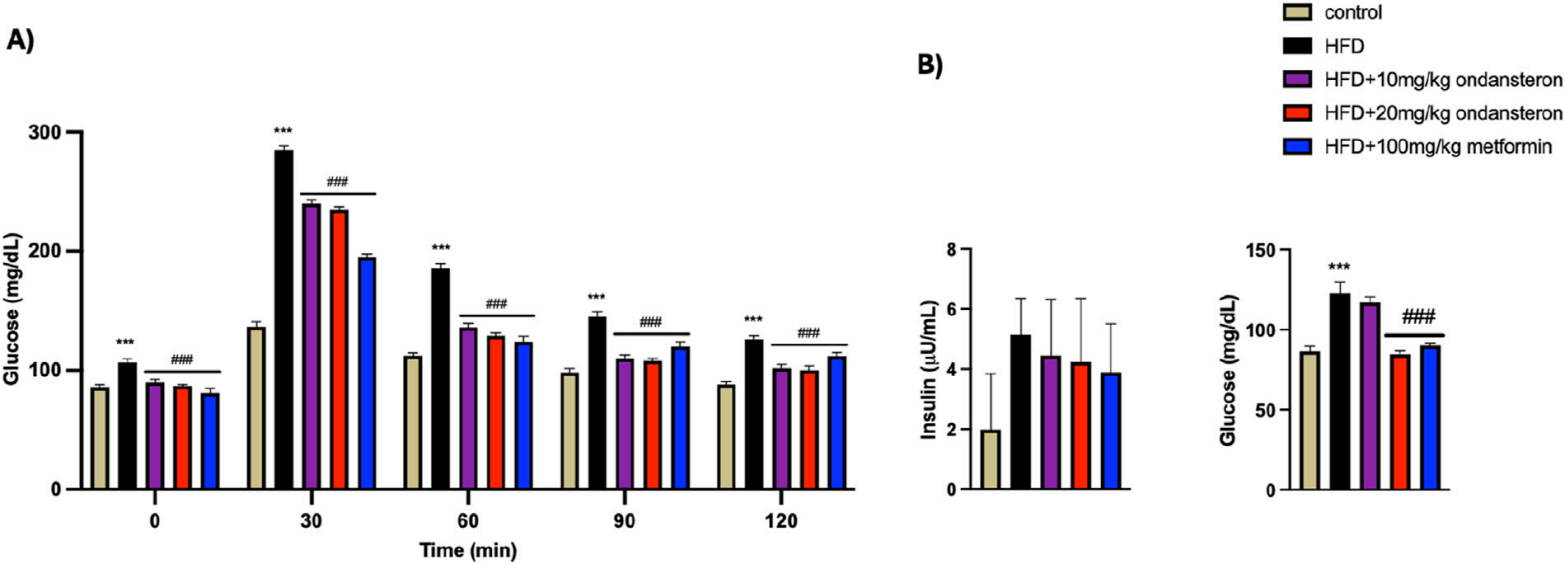
Bar graph representing blood glucose levels at different time intervals (0–120 min). The HFD group shows increased glucose concentration both in fed **(A)** and fasting states **(B)**, but the treatment groups exhibit lower values, indicating improved glucose intolerance caused by HFD. The effect of HFD and ondansetron treatment on fasting insulin and glucose concentration of rats shows an increase in insulin and glucose load in the body after HFD feeding for 4 months, and ondansetron treatment causes a marked reduction in the concentration of glucose **(B)**. Data are presented as means *±* SEM. One-way ANOVA was used for the analysis of data, followed by a post hoc Bonferroni multiple comparison test using GraphPad Prism 5 software. The symbols * or # represents significant difference values p < 0.05, **or ## represents p < 0.01, and *** or ### represents p < 0.001 values for significant differences. The symbol * shows a significant difference relative to control, and # shows a significant difference relative to the HFD group.

Fluctuations in blood glucose levels over 120 min post-oral glucose administration in various experimental groups were quantified (Figure 6A). Blood glucose levels, measured 30 min after glucose load using a glucometer, were significantly elevated in the HFD group (288.2 ± 3.31 mg/dL) compared to the control group (136.6 ± 4.2 mg/dL). Treatment groups showed reduced glucose levels: 240 ± 3.9 and 235.1 ± 2.65 mg/dl for low (10 mg/kg) and high (20 mg/kg) doses of ondansetron, respectively, while 195.72 ± 4.17 mg/dL was reported for the positive control, i.e., metformin (100 mg/kg). This trend persisted throughout the measurement duration of 120 min (Figure 6A).

HFD increased the fasting insulin levels in control rats. The treatment with either ondansetron or metformin tended to decrease the insulin levels. A gradual decrease could be observed, but the differences were not statistically significant (Figure 6B).

The administration of ondansetron (10 mg/kg and 20 mg/kg) and metformin (100 mg/kg) resulted in significant reductions in serum TG, TC, and LDL cholesterol levels in HFD-fed rats. Specifically, ondansetron at a dose of 20 mg/kg was most effective in lowering TGs (89 ± 3.86 mg/dL), TC (86.78 ± 4.92 mg/dL), and LDL cholesterol (49.86 ± 2.85 mg/dL) compared to the HFD control group (250 ± 2.33 mg/dL, 124 ± 3.34 mg/dL, and 81 ± 5.32 mg/dL, respectively). Ondansetron at a dose of 10 mg/kg also significantly lowered TGs (112 ± 4.22 mg/dL), TC (99.76 ± 3.91 mg/dL), and LDL cholesterol (54.21 ± 3.85 mg/dL) compared to the HFD group. Similarly, metformin (100 mg/kg) also lowered serum TGs (120 ± 5.91 mg/dL), TC (95.93 ± 3.54 mg/dL), and LDL cholesterol (57.88 ± 6.21 mg/dL) compared to the HFD control group. However, none of the treatments had any significant effects on HDL cholesterol levels. These trends suggest that ondansetron and metformin may be effective in managing dyslipidemia associated with HFD.

### Liver enzymes

**FIGURE 7:**
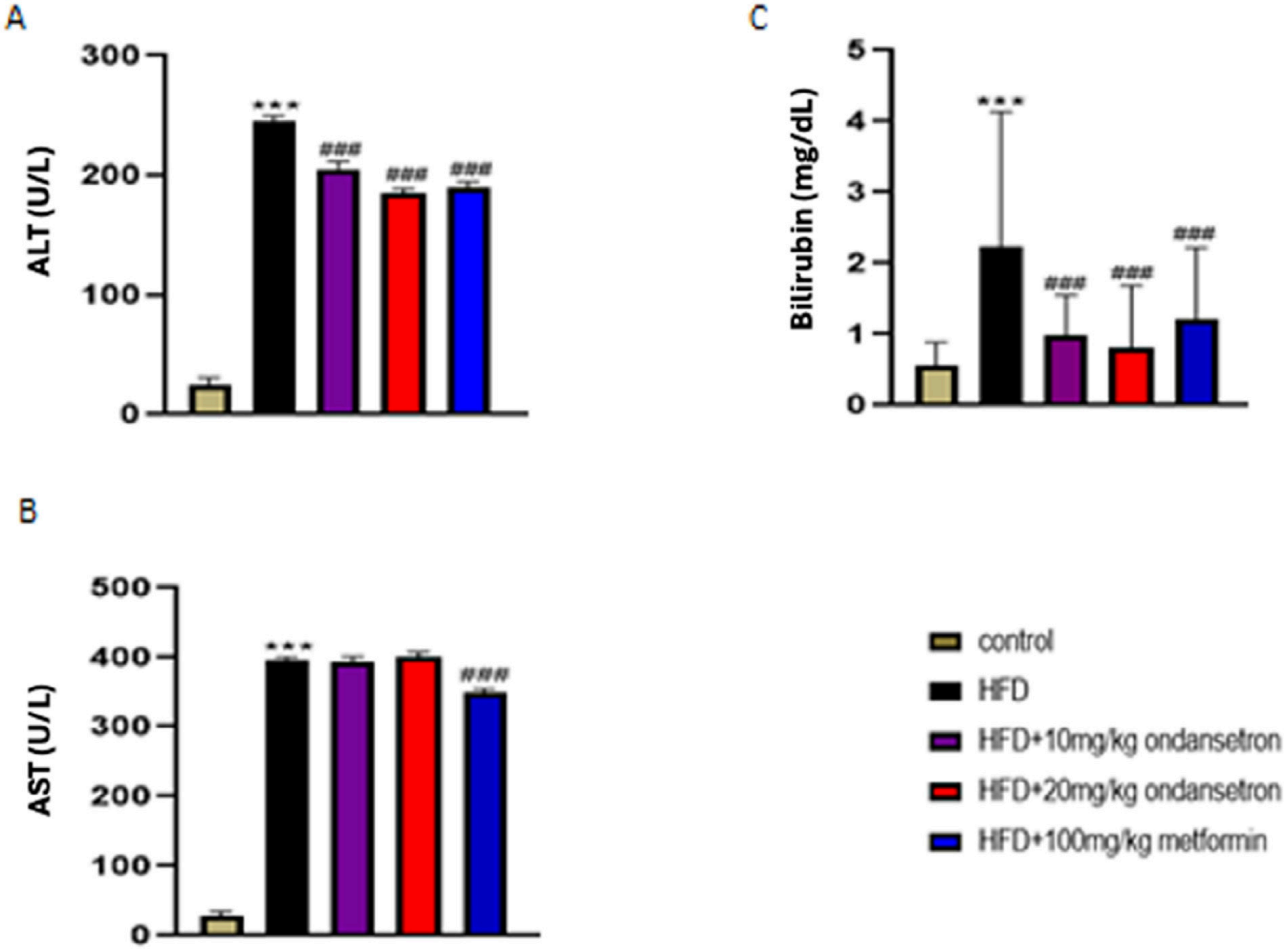
Impact of ondansetron and metformin on liver enzymes. An increase in the levels of hepatic enzymes (ALT, AST, and bilirubin) was observed after HFD feeding. **(A,C)** The treatment or rates with ondansetron for 28 days reduced ALT and bilirubin. **(B)** The treatments did not affect AST levels. Metformin reduced the levels of all three liver enzymes. Data are presented as means ± SEM. One-way ANOVA is used for the analysis of data, followed by a post hoc Bonferroni multiple comparison test using GraphPad Prism 5 software. The symbol * or # represents significant difference values p < 0.05, ** or ## represents p < 0.01, and *** or ### represents p < 0.001 values for significant differences. The symbol * shows a significant difference relative to control, and # shows a significant difference relative to the HFD group.

Liver function tests (LFTs) were conducted to assess the impact of HFD on liver health and the efficacy of treatments with ondansetron and metformin. The HFD group exhibited significant liver damage, indicated by elevated levels of ALT, AST, and bilirubin, compared to the control group (Figure 7). ALT levels significantly increased in the HFD group (245.45 ± 4.6 U/L) compared to the control group (24.45 ± 6.3 U/L). Treatment with ondansetron at 10 mg/kg and 20 mg/kg reduced ALT levels to 205 ± 7.2 U/L and 189 ± 4.3 U/L, respectively (P < 0.001 vs HFD). Metformin (100 mg/kg) showed a more substantial reduction in ALT levels to 190 ± 5.8 U/L (P < 0.001 vs HFD). AST levels were significantly higher in the HFD group (396 ± 6.21 U/L) compared to the control group (28 ± 6.87 U/L) (P < 0.001). Ondansetron treatment did not significantly affect AST levels. However, metformin treatment resulted in a significant decrease in AST levels to approximately 348 ± 8.94 U/L (P < 0.001 vs HFD). Bilirubin levels in the HFD group were markedly elevated (2.34 ± 1.65 mg/dL) compared to the control group (0.5 ± 0.34 mg/dL) (P < 0.001). Ondansetron reduced bilirubin levels to 0.99 ± 0.56 mg/dL and 0.81 ± 0.89 mg/dL at doses of 10 mg/kg and 20 mg/kg, respectively (P < 0.01 vs HFD). Metformin effectively reduced bilirubin levels to 1.21 ± 1.02 mg/dL (P < 0.001 vs HFD).

### Gross and histopathological examination of the liver

**FIGURE 8:**
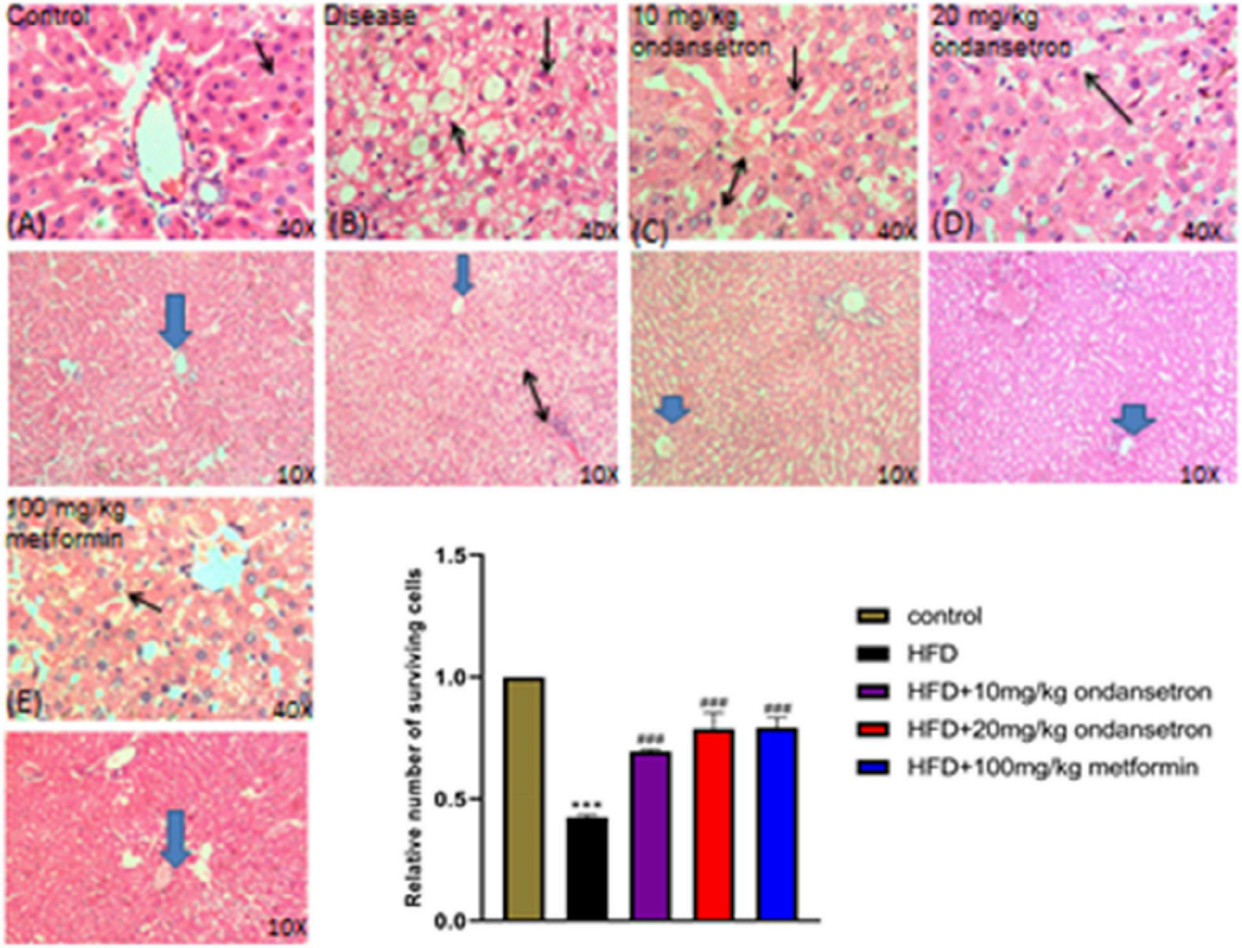
Histological examination of control group and treatment groups. **(A)** Control group shows the typical liver architecture, where the portal tract is at the peripheral side of a classic hepatic lobule uncovering a portal venule (V), and binucleated cells with visible nuclei are present. **(B–E)** In the respective HFD group, dilation of the central vein (arrowhead), cellular infiltration between the hepatocytes (two-sided arrow), and excess of vacuolation (double arrow) are shown, along with fats deposited in the tissues in the disease (HFD) group. The ondansetron groups **(C,D)** show less infiltration (double arrow) and vacuolation. Mild dilation of the central vein (arrowhead) is shown, and most of the liver cells appear normal in shape and size (arrow). Data are presented as means ± SEM. Scale bar = 50 μm; n = 5/group.

After 28 days, the rats were euthanized. The livers were retrieved and rinsed with normal saline. Then, the macroscopic examination of the livers’ physical appearance was conducted in comparison to the control group, the HFD group, and the treatment group. As illustrated in Figure 8, the normal liver (a) and liver from ondansetron only (b) had a typical red, smooth, and shiny appearance. In contrast, the livers obtained from the fatty liver disease group exhibited a yellow color with deeply penetrated spots, along with inflammation (c). Interestingly, the livers from the treatment groups displayed a red–brownish color with smooth surfaces, resembling the control group (d, e, and f).

### Immunohistochemical analysis

Liver tissue sections were stained with H&E and examined under light microscopy at ×10 and ×40 magnifications (Figure 8). The control group displayed normal liver histoarchitecture with well-preserved hepatocytes, identifiable central veins, and no significant cellular damage or fatty infiltration (**A**). The disease group showed extensive hepatic steatosis, ballooning degeneration, and scattered areas of hepatic necrosis (black arrows) (**B**). Ondansetron treatment (10 mg/kg) partially ameliorated disease-induced histopathological changes, reducing hepatic steatosis and modestly preserving the liver architecture, although some hepatocellular ballooning and necrosis were still evident (black arrows) (**C**). Treatment with 20 mg/kg ondansetron demonstrated a more pronounced protective effect, with further reduction in steatosis and nearly normal hepatocyte morphology. Minor residual necrotic areas and ballooning degeneration were observed (black arrows) (**D**). Metformin treatment (100 mg/kg) showed the most significant protective effect, with a marked reduction in lipid accumulation, improved cellular integrity, and minimal evidence of necrosis or other pathological features (**E**). The bar graph quantitatively assessed the relative number of surviving hepatocytes. The disease group exhibited a significant reduction in viable cells compared to controls (P < 0.001). Treatment with 10 mg/kg and 20 mg/kg ondansetron significantly increased hepatocyte survival compared to the disease group (P < 0.001), with the 20 mg/kg dose showing a larger effect. Metformin (100 mg/kg) treatment demonstrated the most substantial improvement in cell survival (P < 0.001), nearly restoring viable cell numbers to control levels.

### Immunohistochemical analysis

The control group exhibited minimal TNF-α immunoreactivity with low staining intensity. In contrast, the disease group showed significantly elevated TNF-α expression, indicating increased pro-inflammatory cytokine levels. Treatment with ondansetron at 10 mg/kg and 20 mg/kg reduced TNF-α staining intensity, with the higher dose showing a more pronounced effect. Metformin at 100 mg/kg substantially reduced the TNF-α expression to levels comparable to those of the control group. Histological analysis revealed minimal COX-2 staining in the control group. The disease group, however, exhibited a marked increase in COX-2 immunoreactivity, indicative of heightened inflammatory responses. Ondansetron treatment at 10 mg/kg and 20 mg/kg reduced COX-2 expression, with the higher dose showing greater efficacy.

**FIGURE 9:**
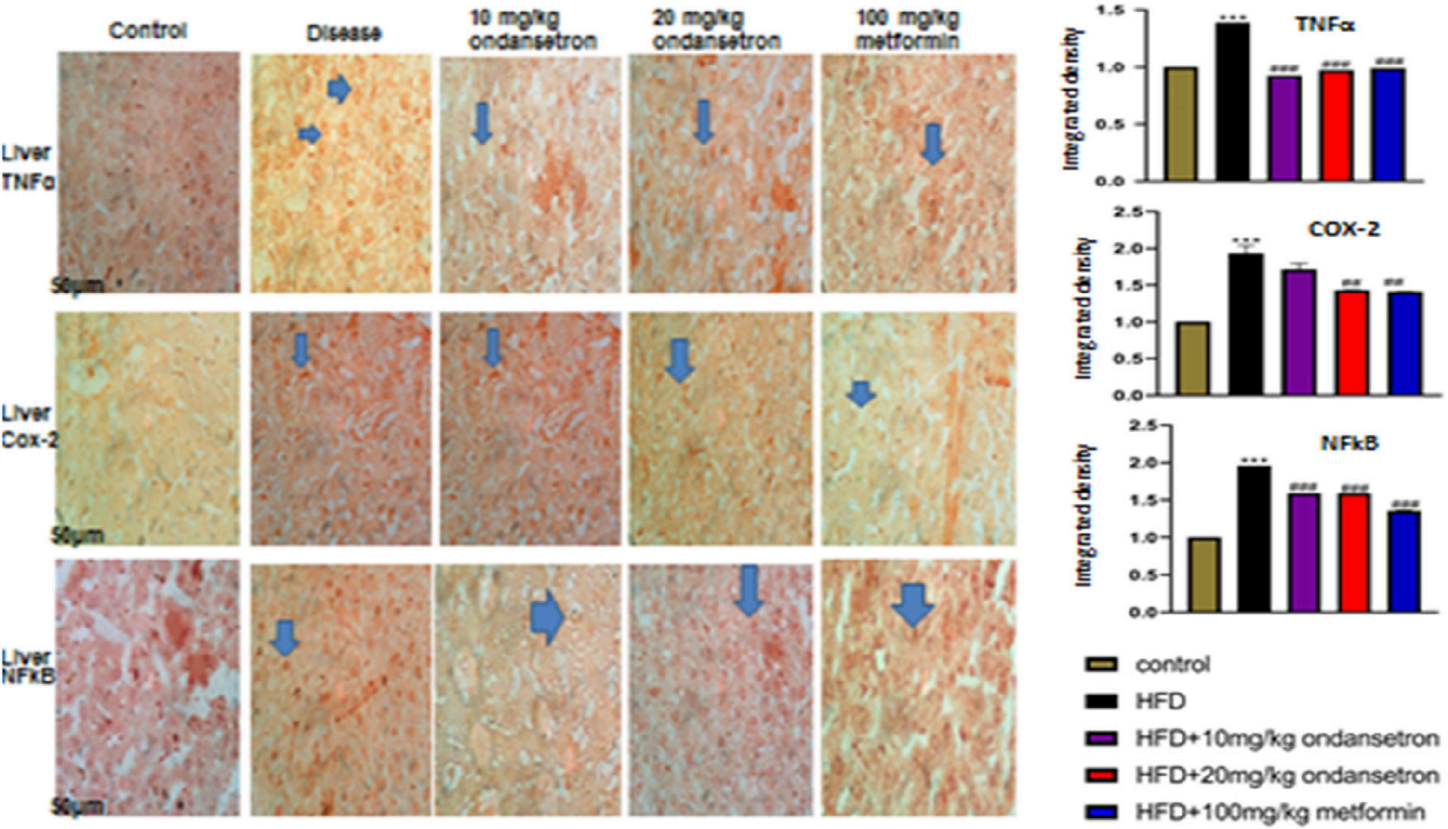
Effects of HFD and ondansetron on pro-apoptotic and pro-inflammatory markers of TNF-α, NF-κB, and COX-2. The ×40 magnification was used for the analysis (n = 5/group). Morphological data were analyzed through ImageJ software. The bar graph represents the relative integrated optical density (IOD) of these inflammatory markers in each group. Data are presented as means *±* SEM. One-way ANOVA is used for the analysis of data, followed by a post hoc Bonferroni multiple comparison test using GraphPad Prism 5 software. The symbols * or # represents significant difference values p < 0.05, **or ## represents p < 0.01, and *** or ### represents p < 0.001 values for significant differences. The symbol * shows a significant difference relative to control, and # shows a significant difference relative to the HFD group.

After administering a dose of 100 mg/kg of metformin, almost all the COX-2 levels were normalized in the control group. Consistent TNF-α and COX-2 patterns were observed with respect to NF-κB staining, as represented in Figure 9. Minimum NF-κB immunoreactivity was observed in the control group, whereas a considerable increase was observed in the staining intensity of the disease group. Significant reduction was observed in NF-κB staining when ondansetron was administered in 10 mg/kg and 20 mg/kg doses, in which the higher dose showed a more enhanced effect. At a dose of 100 mg/kg, metformin caused a clear reduction in NF-κB levels, which is quite similar to the control group. The histological findings were confirmed by conducting the quantitative analysis of integrated optical density (IOD). A prominent increase in the IOD values of COX-2, NF-κB, and TNF-α was observed in the disease group in comparison with the control group (p < 0.001). A prominent decrease in IOD values was observed for all markers when 10 mg/kg and 20 mg/kg doses were administered in a dose-dependent manner. A 100 mg/kg dose of metformin showed a further decrease in the IOD values, which were similar to the control group (p < 0.001).

### Inflammatory mediators in liver tissue

**FIGURE 10:**
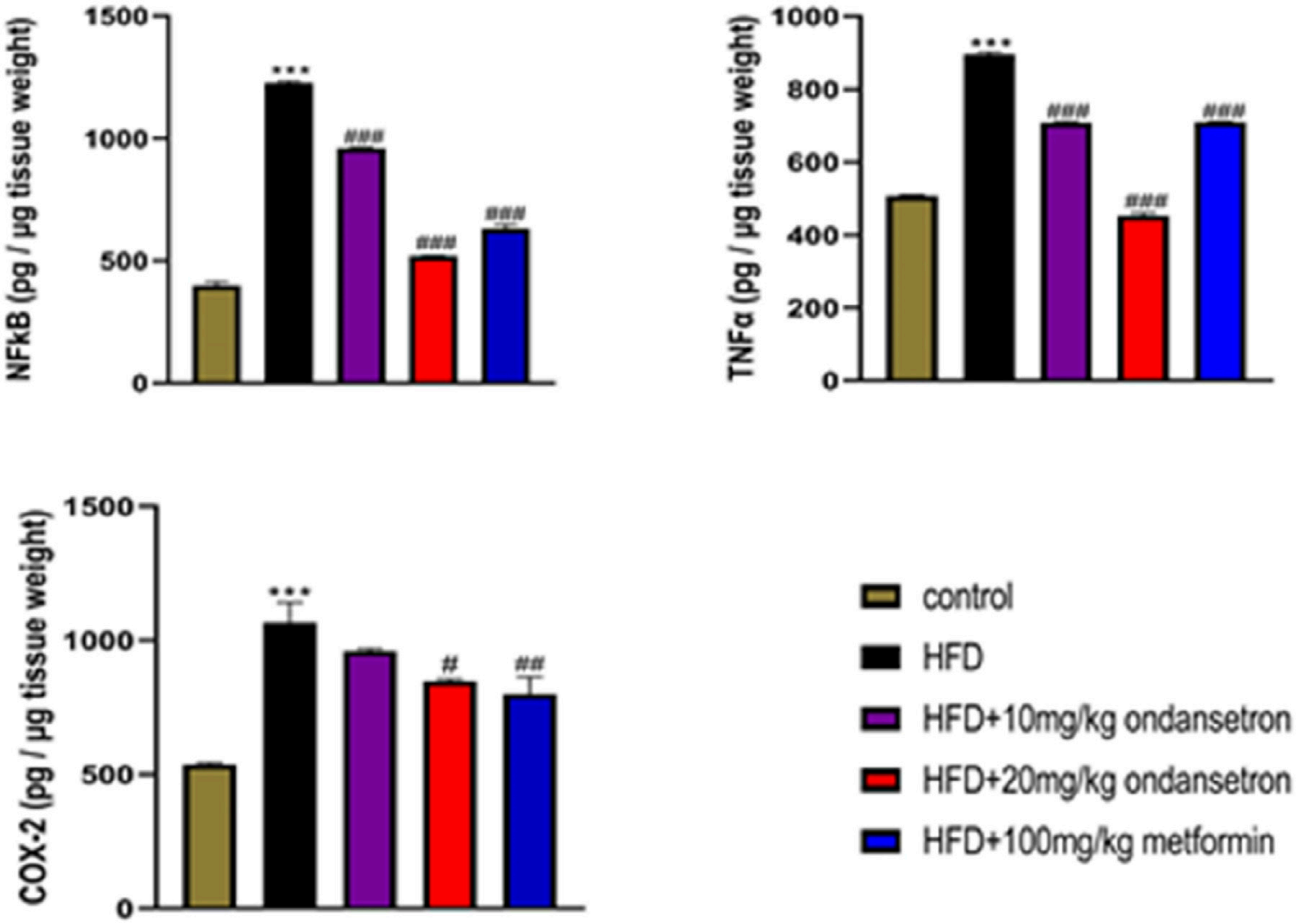
Analyzing the expression of inflammatory markers by ELISA in liver homogenates showed increased levels in the HFD group compared to the chow-fed animals. Ondansetron (20 mg/kg) had a strong anti-inflammatory effect in the livers of HFD-fed rats by reversing the increase in the protein expression. Data were presented as means *±* SEM. One-way ANOVA is used for the analysis of data, followed by a post hoc Bonferroni multiple comparison test using GraphPad Prism 5 software. The symbol * or # represents significant difference values p < 0.05, **or ## represents p < 0.01, and *** or ### represents p < 0.001 values for significant differences. The symbol * shows a significant difference relative to control, and # shows a significant difference relative to HFD.

A marked increase in the expression of inflammatory mediators TNF-α, COX-2, and NF-κB was observed in the liver homogenates of the disease group (899.2 pg/μg, 1,120 pg/μg, and 1,230 pg/μg, respectively) as compared to the control group (504.27 pg/μg, 542.3 pg/μg, and 410 pg/μg, respectively). Interestingly, the treatment of HFD-fed rats with both high and low doses of ondansetron (10 and 20 mg/kg, respectively) significantly reduced the expression of all three pro-inflammatory effectors (708.3 pg/μg and 455.3 pg/μg for TNF-α, 843 pg/μg and 755 pg/μg for COX-2, and 957 pg/μg and 521 pg/μg for NF-κB) (Figure 10).

### Antioxidant enzymes and peroxidation in liver tissue

**FIGURE 11:**
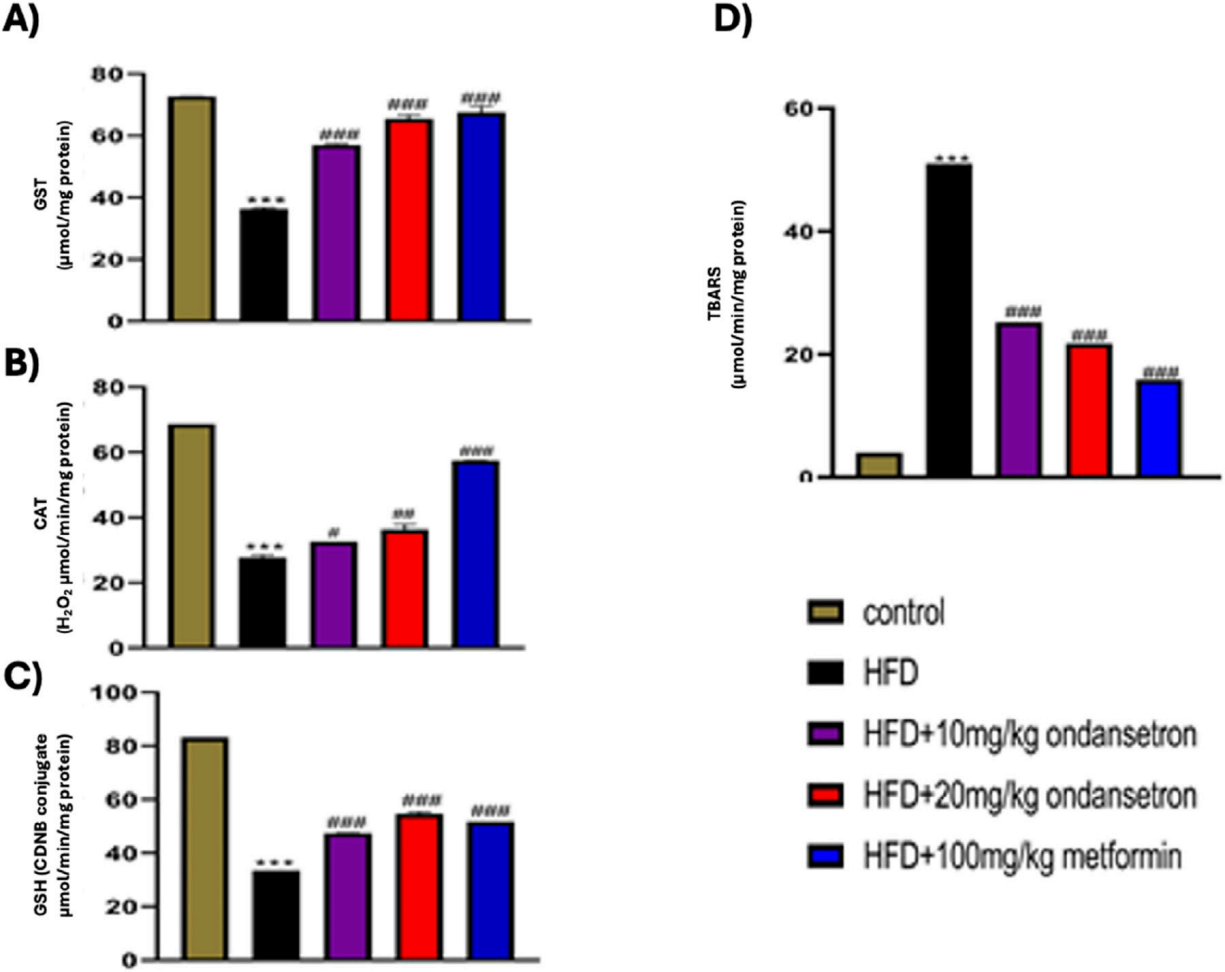
Effect of ondansetron on oxidative stress-related antioxidant enzymes: GST **(A)**, CAT **(B)**, and GSH **(C)**, in addition to TBARS **(D)**. HFD feeding reduced the levels of antioxidant enzymes and TBARS in rat livers. Ondansetron significantly reversed the effects of HFD treatment. Data are presented as means *±* SEM. One-way ANOVA is used for the analysis of data, followed by a post hoc Bonferroni multiple comparison test using GraphPad Prism 5 software. The symbol * or # represents significant difference values p < 0.05, **or ## represents p < 0.01, and *** or ### represents p < 0.001 values for significant differences. The symbol * shows a significant difference relative to control, and # shows significant difference relative to the HFD group.

HFD treatment significantly decreased the levels of both enzymatic and non-enzymatic antioxidants in the liver of rats, as illustrated in Figure 11. The levels of CAT, GST, and GSH were markedly reduced in the HFD group compared to the control group, with values of 28.33 ± 0.34 μmol/min/mg, 36.65 ± 0.65 µmol CDNB conjugate/min/mg, and 33.52 ± 0.86 μmol/mg of protein, respectively (p < 0.001). In contrast, rats treated with ondansetron at doses of 10 mg/kg and 20 mg/kg exhibited a significant increase in the levels of these antioxidant enzymes. Specifically, CAT levels increased to 32.22 ± 0.56 μmol/min/mg and 37.45 ± 0.45 μmol/min/mg (*p* < 0.01 and *p* < 0.001, respectively), GST levels increased to 56.78 ± 0.43 μmol/mg and 64.71 ± 0.56 μmol/mg (*p* < 0.05 and *p* < 0.001, respectively), and GSH levels increased to 47.44 ± 0.78 CDNB conjugate/min/mg and 54.10 ± 0.66 CDNB conjugate/min/mg (*p* < 0.05 and *p* < 0.001, respectively) compared to the HFD group. As shown in Figure 11D, the thiobarbituric acid reactive substance (TBARS) assay indicated that HFD significantly elevated the levels of peroxides to 51.003 ± 0.88 μmol/mg of protein compared to the control group (*p* < 0.001). However, treatment with ondansetron at 10 mg/kg and 20 mg/kg significantly reduced the peroxide levels to 25.245 ± 0.53 and 21.750 ± 0.73 μmol/mg of the protein, respectively (*p* < 0.001). These antioxidant protective effects of ondansetron were comparable to those observed with metformin (100 mg/kg), which also significantly restored the antioxidant enzymatic levels and reduced TBARS.

## Discussion

The current study investigates the candidature of PTP1B as a potential target for diabetes and NAFLD, followed by the elucidation of the therapeutic effectiveness of ondansetron in these conditions. The study employed a multifaceted approach, combining computational analyses, enzyme inhibition assay, and *in vivo* experiments, to unravel the drug’s effects on key metabolic pathways and disease markers. The *in silico* studies provided valuable insights into the molecular interactions of ondansetron with its target protein, PTP1B. It is evident from the pharmacophore modeling and molecular docking studies that effective binding exists between the allosteric site of PTP1B and ondansetron, which depicts the ability of the ligand to regulate enzymatic activity. This finding is further justified from the results of the MD simulation, which suggests that apart from the binding of ondansetron to the protein, it also adds stability to the protein, proving it to be better than its native ligand during various ligand–protein interactions. The results of simulations clearly indicate a strong interaction profile, and high stability highlights the capability of ondansetron to have an influence on regulating PTP1B activity. The drug molecule competitively binds to 5-hydroxytryptamine or the serotonin receptor, thus carrying out a dual pharmacological action (Zhang et al., 2020). Serotonin, a pro-inflammatory mediator, is associated with the symptoms of NAFLD through mTOR signaling (Feng et al., 2022). Specificity of the molecule for PTP1B was, therefore, established through the enzyme inhibition assay. This *in vitro* assay revealed that the PTP1B activity was directly inhibited by ondansetron. It was observed that a dose-dependent inhibitory effect was produced by ondansetron on PTP1B, in which the enzymatic activity decreased with the increase in the dose, as reported in similar studies (Liu et al., 2022). Moreover, the inhibitory potency of suramin, which is used as positive control in the study, was similar to that of ondansetron. It is clear from the results of the enzyme inhibition assay that ondansetron has the ability to inhibit the activity of PTP1B, and this finding is similar to the hypothesis developed from *in silico* studies. The direct action of ondansetron on the inhibition of PTP1B activity contributes to laying down the foundation that this metabolic pathway can be instrumental in treating conditions like NAFLD and diabetes, as proposed in our earlier studies (Agouni et al., 2011; 2014; Abdelsalam et al., 2021).

The studies performed on animals (rats) by developing an HFD- induced obesity model strengthened the outcomes of our *in silico* studies and highlights the treatment potential of ondansetron. The abilities of ondansetron to reduce glucose levels and enhance the sensitivity of insulin are the main factors that contribute to diabetes treatment. Furthermore, the useful impact of ondansetron on improving the lipid profile advocates for its positive role in various mechanisms of lipid metabolism like a prominent decrease in LDL cholesterol, total cholesterol, and serum triglycerides, thereby highlighting its ability to treat dyslipidemia related to metabolic abnormalities (Shinohara et al., 2019). The role of ondansetron in inducing hepatoprotective activity was also investigated. An overall improvement was observed in the architecture of liver cells characterized by a decrease in the liver damage markers, cellular infiltration, steatosis, and necrosis, the hallmarks of liver function recovery (Campo et al., 2018). Moreover, the study also accounts for the anti-inflammatory and antioxidant characteristics of ondansetron. A positive prominent effect on the inflammatory mechanisms was observed, thereby showing reduction in COX-2, TNF-α, and NF-κB levels present in the tissues of liver, which were endorsed by analyzing immunohistochemistry analysis. In addition, the elevated levels of antioxidant enzymes and reduction in peroxidation indicate the ability of ondansetron to produce antioxidant activity, as reported earlier (Özmen and Küfrevıoǧ, 2004). It can be inferred from these findings that ondansetron may have the ability to counteract conditions like oxidative stress and inflammation for therapeutic outcomes related to NAFLD.

The evidence of the study is in coherence with the outcomes of the earlier studies on the inhibitory potential of ondansetron on PTP1B activity in various metabolic disorders. PTP1B came into limelight as a primary target responsible for the progression of abnormal conditions like obesity, insulin resistance, and NAFLD, which is a down-regulator of leptin and insulin pathways (Delibegovic et al., 2009). Defects in insulin signaling appear, which further produce abnormal lipid profile, glucose intolerance, and steatosis of liver (Zabolotny et al., 2008). So, focusing on PTP1B seems to be a positive approach to treat different abnormal metabolic conditions.

PTP1B inhibitors have been incorporated into various preclinical trials, and their effectiveness has been established by exhibiting a reduction in body weight, improvement in insulin sensitivity, and eradication of NAFLD. On the other hand, a number of PTP1B inhibitors have faced some unavoidable complications pertaining to the bioavailability, selectivity, and some serious severe side effects (Hussain et al., 2019). By repurposing ondansetron, which is an FDA-approved drug, this study offers a solution, along with a secure safety profile. It is evident from the *in silico* and *in vivo* results of this study that ondansetron has the ability to bind with PTP1B and can produce positive therapeutic effects. Moreover, the antioxidant and anti-inflammatory actions of ondansetron advocate for its promising effects against NAFLD. Oxidative stress and persistent inflammation are the main factors that contribute to NAFLD development, leading to NASH, which is a more extreme condition involving fibrosis and inflammation of hepatic cells (Friedman et al., 2018). As ondansetron engages in these processes, it can not only deliver a therapeutic effect in metabolic dysfunction but can also play its part in the reversal of NAFLD.

The result of this study highlights the significance of repurposing in developing new approaches for complicated conditions, e.g., NAFLD. There are various merits of repurposing compared to the conventional drug discovery process, which includes less cost and time consumption, along with an authentic safety profile (Li and He, 2023). In preclinical trials against NAFLD models, ondansetron has been identified as a strong inhibitor of PTP1B activity, demonstrating pronounced pharmacological activity that supports its effectiveness to be used in the therapeutic context. Our study, hence, provides convincing evidence for the therapeutic role of ondansetron pertaining to diabetes and NAFLD, indicating that there is more room for improvement in the research domain to understand its mechanism of action and assess its effectiveness/safety profile for further implementation. The anti-diabetic, antioxidant, and anti-inflammatory actions of ondansetron are the primary target areas for future investigation. The findings of this study highlight that the inhibition of PTP1B can result in producing novel therapeutic effects in conditions like type 2 diabetes and NAFLD.

## Conclusion

Studies related to the NAFLD and NASH models should also be conducted to reveal the role of ondansetron. It is important to carry out well-planned therapeutic trials that can be instrumental in highlighting the action of ondansetron in conditions like diabetes. When the results of therapeutic trails are justified by ondansetron, it can be used as a drug of choice for treating different metabolic disorders. This unique property of ondansetron to target different pathways sets it apart from other drug molecules. The role of ondansetron in the pathophysiology of diseases and a known safety profile make it an ideal candidate for drug repurposing. The successful translation of ondansetron into clinical practice can have a significant impact on the management of diabetes and NAFLD, offering hope for improved patient outcomes and reduced disease burden.

## Data Availability

The raw data supporting the conclusions of this article will be made available by the authors, without undue reservation.
