# Supplementary material for: The effects of ondansetron on diabetes and high-fat diet-induced liver disease: a critical role for protein tyrosine phosphatase 1B
Source: Front Pharmacol. 2025 Apr 28;16:1565628. doi: 10.3389/fphar.2025.1565628 (PMC12066537; doi:10.3389/fphar.2025.1565628)
Supplement: Supplementary file 1 [file DataSheet1.docx]

**SUPPLEMENTARY FILE**

The Effects of Ondansetron on Diabetes and High Fat Diet-Induced Liver Disease: A Critical Role for Protein Tyrosine Phosphatase 1B

Fawad Naeem, Maryam Aqeel, Muhammad Ammar Zahid, Mustafeez Mujtaba Babar, Fawad Ali Shah, Abdelali Agouni, Sohaib Zafar Malik


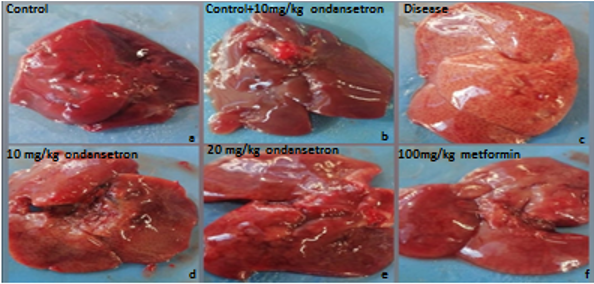


**Figure S1.** Gross examination of the liver from normal, HFD-fed, and treatment groups are shown indicating shiny, reddish liver with normal liver architecture in the control group (a) and to some extent the treated groups as well (d, e, f), but the diseased liver is showing pronounced inflammation with yellow patches and signs of liver damage can also be observed clearly (c).

**Table S1.** Top 20 screened compounds/conformations against the developed Pharmacophore model and their respective phase screen scores (Higher is better).

| **DATABASE_ID** | **GENERIC_NAME** | **PHASE SCREEN SCORE** |
| --- | --- | --- |
| DB00904 | Ondansetron | 1.943 |
| DB08103 | 2,6-dibromo-4-phenoxyphenol | 1.862 |
| DB02583 | N6-(2,5-Dimethoxy-Benzyl)-N6-Methyl-Pyrido[2,3-D]Pyrimidine-2,4,6-Triamine | 1.856 |
| DB02583 | N6-(2,5-Dimethoxy-Benzyl)-N6-Methyl-Pyrido[2,3-D]Pyrimidine-2,4,6-Triamine | 1.856 |
| DB06150 | Sulfadimethoxine | 1.856 |
| DB01838 | 6,4'-Dihydroxy-3-Methyl-3',5'-Dibromoflavone | 1.855 |
| DB02583 | N6-(2,5-Dimethoxy-Benzyl)-N6-Methyl-Pyrido[2,3-D]Pyrimidine-2,4,6-Triamine | 1.854 |
| DB00904 | Ondansetron | 1.852 |
| DB02427 | 2,4-Diamino-6-[N-(2',5'-Dimethoxybenzyl)-N-Methylamino]Quinazoline | 1.852 |
| DB02427 | 2,4-Diamino-6-[N-(2',5'-Dimethoxybenzyl)-N-Methylamino]Quinazoline | 1.851 |
| DB02427 | 2,4-Diamino-6-[N-(2',5'-Dimethoxybenzyl)-N-Methylamino]Quinazoline | 1.851 |
| DB04215 | CRA_9076 | 1.851 |
| DB08465 | 2-(3-AMINO-2,5,6-TRIMETHOXYPHENYL)ETHYL 5-CHLORO-2,4-DIHYDROXYBENZOATE | 1.845 |
| DB09151 | Flutemetamol (18F) | 1.844 |
| DB15058 | Flutemetamol | 1.844 |
| DB08204 | 3-DIPHENOL-6-NITRO-3H-BENZO[DE]ISOCHROMEN-1-ONE | 1.844 |
| DB13437 | Medazepam | 1.843 |
| DB07694 | 2,5-dichloro-N-(3,5-dibromo-4-hydroxyphenyl)benzamide | 1.841 |
| DB02277 | 1-(5-Tert-Butyl-2-Methyl-2h-Pyrazol-3-Yl)-3-(4-Chloro-Phenyl)-Urea | 1.84 |
| DB07354 | 2,3-DIMETHOXY-12H-[1,3]DIOXOLO[5,6]INDENO[1,2-C]ISOQUINOLIN-6-IUM | 1.839 |

**Table S2.** Top 20 screened compounds for the protein PTP1B and their relative docking scores.

| **DATABASE_ID** | **GENERIC_NAME** | **DOCKING SCORE** |
| --- | --- | --- |
| DB13225 | Dibenzepin | -9.417 |
| DB01059 | Norfloxacin | -9.318 |
| DB13228 | Flosequinan | -9.245 |
| DB13931 | Netarsudil | -9.202 |
| DB00543 | Amoxapine | -9.023 |
| DB00904 | Ondansetron | -8.924 |
| DB05271 | Rotigotine | -8.896 |
| DB00487 | Pefloxacin | -8.884 |
| DB00623 | Fluphenazine | -8.844 |
| DB00776 | Oxcarbazepine | -8.814 |
| DB05039 | Indacaterol | -8.791 |
| DB09084 | Benzydamine | -8.699 |
| DB00457 | Prazosin | -8.692 |
| DB13680 | Naftazone | -8.64 |
| DB00253 | Medrysone | -8.629 |
| DB08907 | Canagliflozin | -8.624 |
| DB01084 | Emedastine | -8.578 |
| DB14575 | Eslicarbazepine | -8.565 |
| DB00247 | Methysergide | -8.546 |
| DB13256 | Clothiapine | -8.532 |
